# Analysis of the Expression, Secretion and Translocation of the *Salmonella enterica* Type III Secretion System Effector SteA

**DOI:** 10.1371/journal.pone.0026930

**Published:** 2011-10-27

**Authors:** Elena Cardenal-Muñoz, Francisco Ramos-Morales

**Affiliations:** Departamento de Genética, Facultad de Biología, Universidad de Sevilla, Sevilla, Spain; Indian Institute of Science, India

## Abstract

Many Gram-negative pathogens possess virulence-related type III secretion systems. *Salmonella enterica* uses two of these systems, encoded on the pathogenicity islands SPI-1 and SPI-2, respectively, to translocate more than 30 effector proteins into eukaryotic host cells. SteA is one of the few effectors that can be translocated by both systems. We investigated the conditions affecting the synthesis of this effector, its secretion to culture media and its translocation into host cells. Whereas *steA* was expressed under a wide range of conditions, some factors, including low and high osmolarity, and presence of butyrate, decreased expression. SteA was efficiently secreted to the culture media under both SPI-1 and SPI-2 inducing conditions. The kinetics of translocation into murine macrophages and human epithelial cells was studied using fusions with the 3xFLAG tag, and fusions with CyaA from *Bordetella pertussis*. Translocation into macrophages under non-invasive conditions was mainly dependent on the SPI-2-encoded type III secretion system but some participation of the SPI-1 system was also detected 6 hours post-infection. Interestingly, both type III secretion systems had a relevant role in the translocation of SteA into epithelial cells. Finally, a deletion approach allowed the identification of the N-terminal signal necessary for translocation of this effector. The amino acid residues 1–10 were sufficient to direct translocation into host cells through both type III secretion systems. Our results provide new examples of functional overlapping between the two type III secretion systems of *Salmonella*.

## Introduction

Many pathogenic Gram-negative bacteria rely on type III secretion systems (T3SS) for their interaction with the host. A type III secretion apparatus, also called injectisome or molecular needle [1], is a complex structure, functionally and structurally related to the flagella assembly system, consisting of at least 20 different subunits, that spans the inner membrane, the periplasmic space, and the outer membrane of the Gram-negative bacteria, and the cell membrane of the host [2]. This system allows delivery into the eukaryotic host cells of effector proteins that direct the different stages of the infection at the cellular level.

*Salmonella* is a genus of Gram-negative bacteria that belongs to the family Enterobacteriaceae. Salmonellae are facultative intracellular parasites that can infect a wide variety of animals causing different diseases, from localized intestinal infection to severe systemic disease, depending on the *Salmonella* serovar and the host. *Salmonella enterica* possesses two distinct T3SS, T3SS1 and T3SS2, that are encoded by genes located in two different *Salmonella* pathogenicity islands: SPI-1 and SPI-2, respectively. Function and expression conditions for both systems are different (reviewed in [3]). It is suggested that T3SS2 is a more recently acquired trait because it is not present in another species of the genus: *S. bongori* [4]. The T3SS1 is necessary for the invasion of non-phagocytic cells [5], [6], whereas the T3SS2 is induced after invasion and is essential for survival and replication within macrophages [7]. However, some overlap exists and effectors from both systems are involved in the biogenesis of the *Salmonella*-containing vacuole, a modified phagosome that is the intracellular niche for this pathogen [8], [9], [10], [11] (reviewed in [12]).

More than 30 effectors can be translocated by T3SS1 or T3SS2 into the host cell [13]. They manipulate a number of key host cellular functions, including signal transduction, membrane trafficking and pro-inflammatory immune responses. The related flagella system also contributes to virulence: aside from the effect of flagellar-based motility in the invasiveness of *Salmonella* [14], flagellin is translocated into the cytosol by the T3SS1 of *Salmonella*-infected macrophages and results in activation of the inflammasome and caspase-1-mediated cell death (pyroptosis) [15], [16], [17].

Many *Salmonella* effectors are encoded inside SPI-1 or SPI-2. Most of them are co-expressed with T3SS1 or with T3SS2 and are secreted only through their cognate secretion system. Interestingly, however, some of the effectors encoded outside the two main islands are known to be secreted by both T3SS1 and T3SS2, including SlrP [18], [19], [20], SopD [21], SpvC [22], SspH1 [23], SteA, and SteB [24] (reviewed in [13]) and the recently identified GtgE, SpvD, and SteE [25].

Translocation of *Salmonella* effectors can be studied infecting cultures of different cell lines. Invasive bacteria (expressing T3SS1) are used to infect non-phagocytic cell lines, like epithelial human HeLa cells. Phagocytic lines, like J774 and RAW264.7 macrophages, can also be infected in the same conditions but, since invasive *Salmonella* trigger a rapid form of cell death called pyroptosis in macrophages [26], infections of several hours requires the use of non-invasive bacteria. Interestingly, the expression of T3SS1 and T3SS2 and translocation of effectors change with the conditions used for cultivation of bacteria before infection, with the time post-infection, and with the host cell line [27]. While fractionation and immunoblot with antibodies raised against the effectors or against small tags can be used to analyze translocation, an alternative is the generation of fusions with a fragment of the gene *cyaA* from *Bordetella pertussis* encoding the catalytic domain of a calmodulin-dependent adenylate cyclase. This enzyme converts cellular ATP in cyclic AMP (cAMP) in the presence of calmodulin. Because calmodulin is present in eukaryotic host cells, but not in bacteria, translocation of one of these fusions would be detected as an increase in the level of cAMP in a culture of infected cells [28].

In vitro synthetic growth media can also be used to imitate, to some extent, the in vivo environments. Secretion of effectors to the supernatant of liquid bacterial cultures can then be analyzed by immunoblot. Optimal expression of T3SS1 can be achieved at the end of the exponential growth phase in bacteria cultured in rich media with low aeration and high concentration of NaCl [29], [30]. T3SS2 expression is obtained in acidic minimal media with low phosphate and low magnesium concentration [29], [31], conditions found in the *Salmonella* containing vacuole. T3SS2-dependent secretion can be increased by using strains lacking a complex of SPI-2-encoded proteins SsaM, SpiC, and SsaL, or exposing wild-type bacteria to pH 7.2 after growth at pH 5 [25], [32].

SteA is a poorly characterized T3SS substrate that was identified in a screen to find genes encoding effectors in *S. enterica* serovar Typhimurium [24]. The screen was based on the CyaA system described above. A library of translational fusions between *Salmonella* chromosomal genes and a fragment of the gene *cyaA* from *B. pertussis* was used to infect cultures of J774 macrophages. When these cells were infected for 8 h under non-invasive conditions (stationary-phase bacteria), translocation of SteA depended on T3SS2. However, when using invasive bacteria, SteA was also translocated in a T3SS1-dependent manner into J774 infected for 1 h. A *steA* null mutant showed a three-fold disadvantage in mouse spleen colonization. SteA localized to the *trans*-Golgi network in both transfected and infected human epithelial HeLa cells and to *Salmonella*-induced membrane tubules containing trans-Golgi markers [24], [33].

In this work we focus on SteA as a T3SS1 and T3SS2 effector and we study the conditions that modulate its expression, its secretion to culture media, and its translocation into eukaryotic cells. In addition, we identify the sequences in SteA that are important for secretion and translocation through both systems.

## Results

### Expression of steA under different conditions

SteA is one of the few T3SS *Salmonella* effectors that are secreted by both T3SS1 and T3SS2. During infection *Salmonella* encounters different environments that require the expression of one or the other T3SS. Since both systems are optimally expressed in different conditions, it was interesting to study the expression of *steA* in relation with these conditions. For this purpose, two chromosomal *steA* fusions were generated: a *lacZ* fusion, and a 3xFLAG fusion.

**Figure 1:**
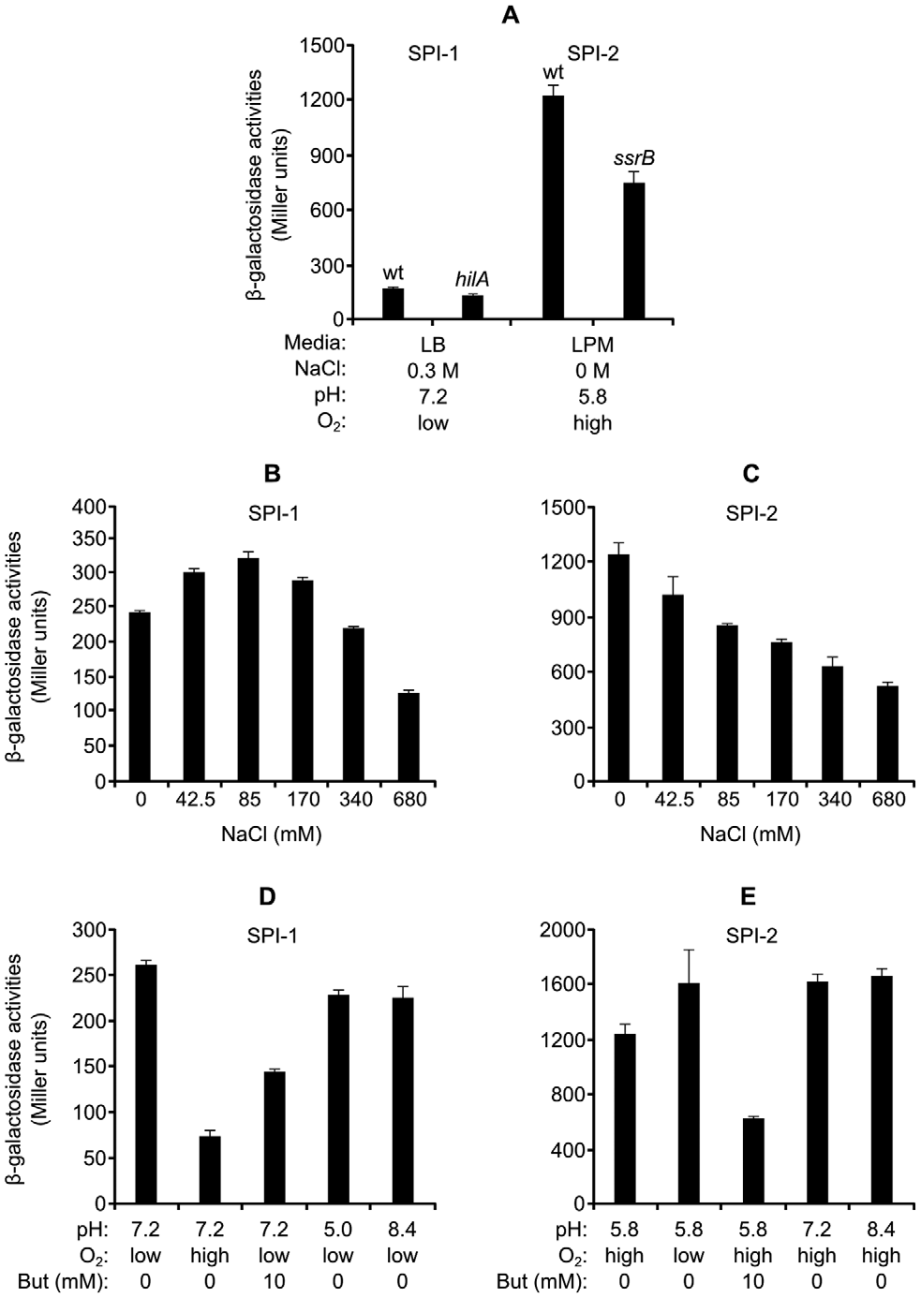
Expression of *steA* in different conditions. Expression levels were monitored with a chromosomal *steA::lacZ* fusion. For optimal SPI-1-inducing conditions, the *S. enterica* strains carrying the fusions were incubated overnight at 37°C without shaking in LB with 0.3 M NaCl. For optimal SPI-2-inducing conditions, the same strains were incubated overnight at 37°C with shaking in LPM. Means and standard deviations from duplicate experiments are represented. (A) β-galactosidase activities were measured in wild-type (wt) and *hilA* backgrounds from cultures in SPI-1-inducing conditions, and in wild-type and *ssrB* backgrounds from cultures in SPI-2-inducing conditions. SPI-1 (B) or SPI-2-inducing (C) cultures with different concentrations of NaCl were used to test the effect of osmolarity. 1 mM proline was added as osmoprotectant to prevent osmotic shock. The effects of pH, oxygen limitation and butyrate (But) on the expression of *steA* were tested under SPI-1 (D) and SPI-2-inducing conditions (E).

The *lacZ* fusion allowed the quantification of expression measuring β-galactosidase activities (Fig. 1). The initial comparison between cultures of the same strain grown overnight at 37°C with low aeration in rich medium (LB) with 0.3 M NaCl or in minimal medium with low phosphate, low pH, and low magnesium (LPM, pH 5.8)), indicated that the expression of *steA* was much higher in LPM (Fig. 1A). The first set of conditions induces optimal expression of the SPI-1 [29], [30] genes whereas LPM mimics the environment that the bacteria find inside the eukaryotic cells in the *Salmonella*-containing vacuole (SCV) and induces the expression of the SPI-2 regulon [34]. This result indicates that *steA* is optimally expressed in coordination with the T3SS2. Key positive regulators of the expression of SPI-1 and SPI-2 are the transcription factors HilA and SsrB, respectively. As seen in Fig. 1A, the expression of *steA* was only slightly reduced by a *hilA* mutation (measured under SPI-1-inducing conditions) or an *ssrB* mutation (under SPI-2-inducing conditions).

Upon an oral infection *Salmonella* finds environmental conditions including acidity, hyperosmolarity and hypoxia and these factors regulate the expression of T3SS1 [35], therefore these conditions were tested in vitro using the LB medium. The same factors were also tested in LPM. High osmolarity and oxygen limitation are used in vitro to induce expression of SPI-1 genes. Expression of *steA* was induced in low oxygen (Fig. 1D and E) but was repressed by high osmolarity (Fig. 1B and C) and this repression was not due to osmotic shock, since the results were obtained in the presence of 1 mM proline used as osmoprotectant. SPI-1 genes are down-regulated after *Salmonella* exposure to butyrate, which is one of the fermentation products found in the intestine. The same is true for *steA* (Fig. 1D and E). Most SPI-1 genes are presumably not expressed in the stomach due to acidity whereas the increase of pH in the intestine could induce their expression. Expression of *steA*, however, was not affected by changes in pH between 5 and 8.4, both in LB (Fig. 1D) or LPM (Fig. 1E). This result indicated that pH is not one of the factors inducing higher expression of *steA* in LPM. A general conclusion of these experiments is that some of the conditions (but not all) that lead to increased expression of SPI-1 genes also up-regulate *steA*.

**Figure 2:**
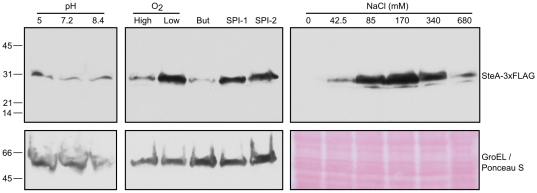
Levels of SteA protein in different conditions. Extracts from an *S. enterica* serovar Typhimurium 14028 (wild-type strain) derivative expressing 3xFLAG-tagged SteA were resolved by 4–15% gradient SDS-PAGE. Immunoblotting was performed with a monoclonal anti-FLAG antibody (upper panels). The conditions tested were: LB with different pH, high or low oxygen (LB cultures with or without shaking, respectively), LB with 10 mM butyrate (But), SPI-1 conditions (LB with 0.3 M NaCl, 16 h at 37°C, without shaking), SPI-2 conditions (LPM pH 5.8), and LB with different concentrations of NaCl, as indicated. Anti-GroEL antibodies were used as loading control (lower panels). Ponceau S red staining was used as loading control when testing the effect of osmolarity since GroEL expression is affected by changes in NaCl concentration. Molecular mass markers, in KDa, are indicated on the left.

The 3xFLAG fusion permitted detection of C-terminally tagged SteA by Western blotting against the FLAG epitope. This fusion was used to monitor the expression of *steA* at the protein level (Fig. 2). The results partially confirmed those obtained with the *lac* fusion: acidic pH slightly increased expression in LB with shaking, but the highest expression was obtained in LB with low aeration and in LPM (SPI-2 conditions). Good expression was also obtained in SPI-1 conditions (cultures in LB 0.3 M NaCl, without aeration). However, the optimal concentration of NaCl in LB cultures was 170 mM. It is interesting to note that the level of SteA protein decreased both at high and low osmolarity, in contrast with the results obtained with the *lac* fusion, suggesting some kind of post-translational regulation.

### Secretion of SteA to the culture media

**Figure 3:**
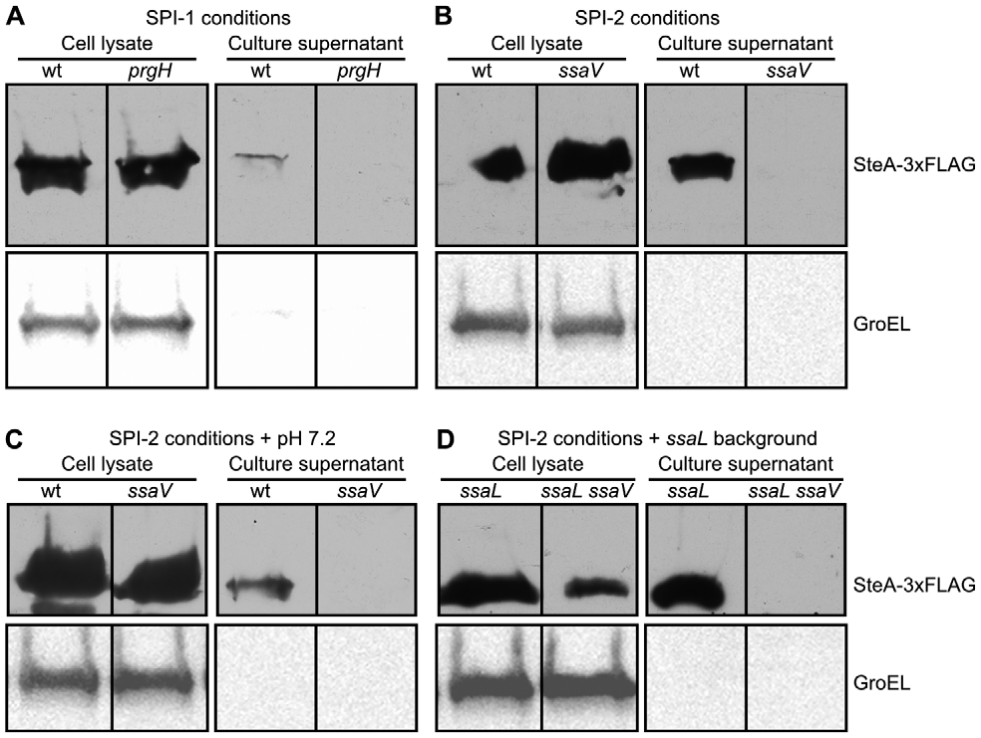
Secretion of SteA to the culture media. The presence of SteA-3xFLAG in cell lysates (from 10^8^ cells) and culture supernatants (from 2.5×10^8^ cells) was analyzed by immunoblotting with anti-FLAG antibodies as described in the Materials and Methods section. The immunoblots were probed for GroEL as a loading control for the cell lysates and to test unspecific leakage of proteins into the supernatants. All the strains used produced a 3xFLAG version of SteA and were derivatives of the wild-type strain 14028 (wt), a *prgH* mutant (lacking the T3SS1) and an *ssaV* mutant (lacking the T3SS2). Bacteria were grown at 37°C using the following conditions: (A) SPI-1 conditions (LB with 0.3 M NaCl, overnight without shaking); (B) SPI-2 conditions (LPM pH 5.8, 24 h with shaking); (C) SPI-2 conditions followed by shifting to pH 7.2 and 1 h incubation; (D) SPI-2 conditions in an *ssaL* background.

Specific media and culture conditions have been used to attain optimal expression of T3SS1 or T3SS2 and to study in vitro secretion of different effectors using these systems. As previously mentioned, overnight cultures in LB with 0.3 M NaCl, without shaking (SPI-1 conditions), are optimal for expression of T3SS1, whereas maximal expression of T3SS2 is obtained in LPM at pH 5.8 (SPI-2 conditions). Secretion of SteA was tested in both conditions using the strain bearing the SteA-3xFLAG fusion. Culture supernatants were concentrated and analyzed by Western blot using anti-FLAG antibodies. Secretion was observed under both conditions (Fig. 3A and 3B) and, as expected, secretion under SPI-1 conditions was dependent on T3SS1 since it was no longer observed in a *prgH* background (PrgH is an essential component of T3SS1), and secretion under SPI-2 conditions was dependent on T3SS2 since it was no longer observed in a *ssaV* background (SsaV is an essential component of T3SS2). It was recently reported that some T3SS2 effectors are more efficiently secreted to the culture media from mutant strains lacking a complex of SPI-2-encoded proteins SsaM, SpiC and SsaL. In addition, wild-type bacteria exposed to pH 7.2 after growth at acidic pH also showed increased secretion of these effectors due to dissociation of the SsaM/SpiC/SsaL complex [32]. On these grounds, we tried these conditions for secretion of SteA, but neither of them improved secretion of this effector through the T3SS2 (Fig. 3C and 3D).

### Translocation of SteA into RAW264.7 macrophages

**Figure 4:**
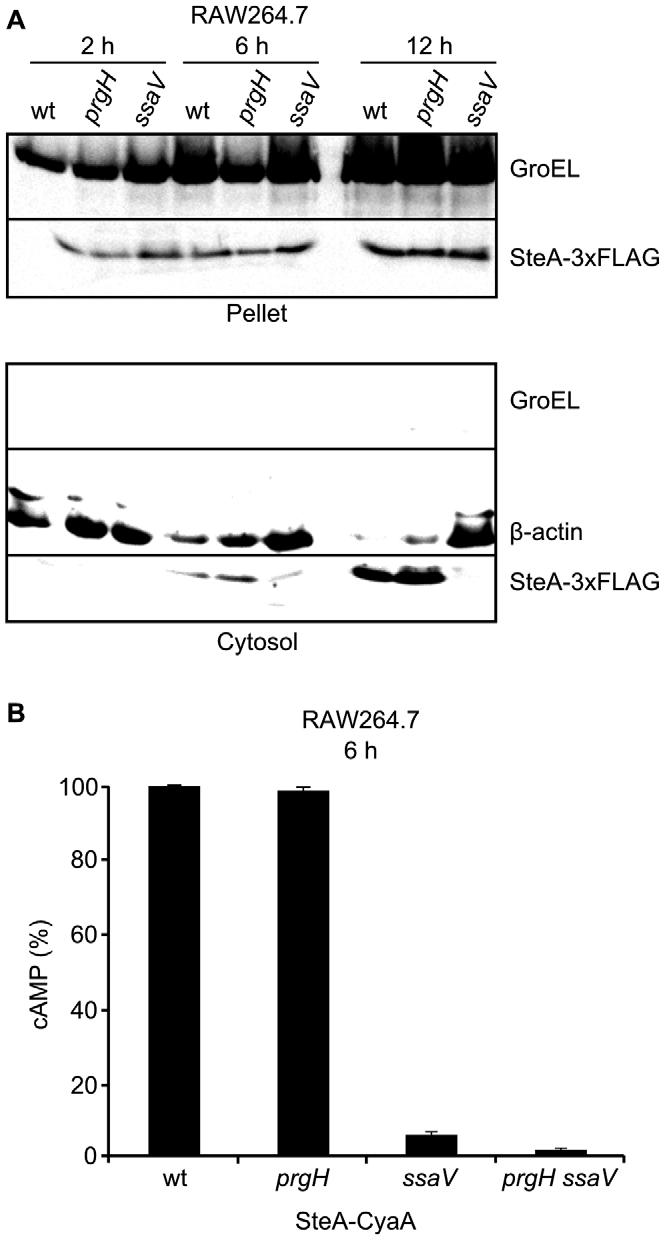
Translocation of SteA into RAW264.7 cells. (A) RAW264.7 cells were infected with *Salmonella* strains expressing SteA-3xFLAG that were derivatives of the wild-type strain (wt), the *prgH* mutant (lacking T3SS1), or the *ssaV* mutant (lacking T3SS2). 2 h, 6 h and 12 h post-infection cells were lysed with 0.1% Triton and submitted to centrifugation. The pellets and the filtered and concentrated supernatants (cytosol) were analyzed by immunoblotting. The membranes were cut in three parts. The top was incubated with anti-GroEL antibodies as a control of contamination of the cytosol fraction with non-secreted bacterial proteins. The middle was incubated with antibodies against the cytosolic protein β-actin to measure the number of eukaryotic cells that was present in each condition. The bottom was incubated with anti-FLAG antibodies to detect the SteA protein that was present in the intracellular bacteria (pellet) and that was translocated into the cytosol of the eukaryotic cells (cytosol). (B) Macrophages RAW264.7 were infected with *S. enterica* serovar Typhimurium wild-type, *prgH*, *ssaV*, or *prgH ssaV* mutant strains, all of them harboring the plasmid pIZ1886 that expresses full length SteA in fusion with the catalytic domain of CyaA from *B. pertussis*. To analyze translocation, the level of cAMP was measured 6 h post-infection as described in the Materials and Methods section. It is expressed as % of the maximum level of cAMP that was measured per intracellular bacterium.

Next, we analyzed the kinetics of translocation of SteA into the eukaryotic host cells using well-established cell lines. First, we infected cultures of the murine macrophage-like cell line RAW264.7 with three strains of *Salmonella* producing SteA-3xFLAG: one strain otherwise wild type, a *prgH* mutant, and an *ssaV* mutant. These bacteria were in non-invasive conditions (grown at 37°C in LB with shaking for 24 h prior to infection) in order to prevent induction of rapid death in the macrophages. 2 h, 6 h and 12 h post-infection, cells were lysed under mild conditions and the eukaryotic cytosol was separated from other components and intact bacteria (pellet) by centrifugation and filtration. The presence of SteA in the pellet and of translocated SteA in the eukaryotic cytosol was investigated by immunoblot with anti-FLAG antibodies (Fig. 4A). Both fractions were also analyzed with anti-GroEL antibodies to have some measure of the survival and growth of bacteria and to get a control for the purity of the cytosol fraction. Anti-β-actin was used to control the relative number of host cells that were present at each time point. As seen in Fig. 4A, translocation of SteA was observed 6 h p.i. but was maximal 12 h after infection. An *ssaV* mutation abolished translocation 12 h p.i., indicating that in these conditions SteA is translocated specifically through the T3SS2. The decreases in the amount of β-actin observed in the infections with the wild-type strain and, to a lesser extent, with the *prgH* mutant, were indicative of the death of the infected cells. This was visually confirmed under the microscope (not shown). In fact, 24 h post-infection most of the cells infected with these strains were dead and it was impossible to evaluate the translocation of SteA (not shown). Translocation 6 h post-infection was only partially suppressed in the *ssaV* background, suggesting the possibility of some translocation through the T3SS1 in these conditions. In order to test this possibility we decided to use a sensitive method based on the generation of a fusion with the catalytic domain of CyaA from *Bordetella pertussis*. This protein is a calmodulin-dependent adenylate cyclase. Calmodulin is not present in bacteria but, upon translocation of a CyaA fusion into the host cell, the adenylate cyclase would be activated by host calmodulin to catalyze the production of cyclic AMP (cAMP). Plasmid pIZ1886 expressing a SteA-CyaA' fusion was introduced in *S. enterica* serovar Typhimurium wild-type strain 14028 and isogenic *prgH* mutant (lacking T3SS1), *ssaV* mutant (lacking T3SS2), and *prgH ssaV* double mutant (lacking both T3SS). These strains were used to infect RAW264.7 cells and the translocation of SteA-CyaA' was monitored 6 h post-infection by measuring the level of cAMP in the cell cultures (Fig. 4B). The results confirm a major role for T3SS2 in the translocation of SteA under these conditions but also support a role for T3SS1.

### Translocation of SteA into HeLa epithelial cells

**Figure 5:**
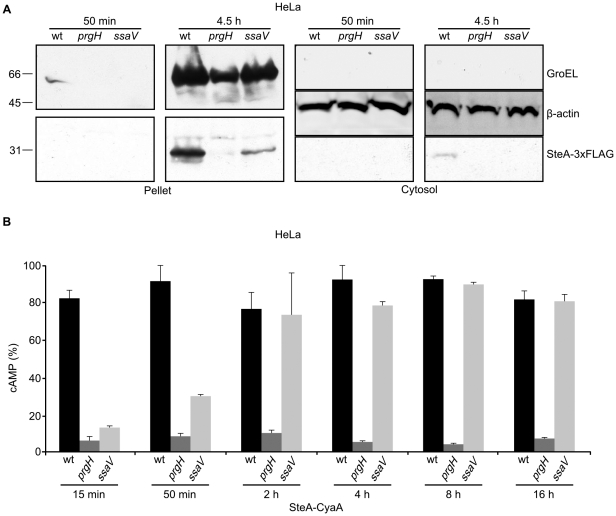
Translocation of SteA into HeLa cells. (A) HeLa cells were infected with *Salmonella* strains expressing SteA-3xFLAG grown under SPI-1 (invasive) conditions and the cells were processed 50 min or 4.5 h post-infection. Immunoblotting was carried out using anti-GroEL (top) and anti-FLAG (bottom) antibodies on the same membranes. (B) Translocation of SteA-CyaA into HeLa cells. HeLa cells were infected with *S. enterica* serovar Typhimurium wild-type, *prgH* or *ssaV* mutant strains, harboring the plasmid pIZ1886 that expresses full length SteA in fusion with the catalytic domain of CyaA from *B. pertussis*. To analyze translocation, the level of cAMP was measured 15 min, 50 min, 2 h, 4 h, 8 h and 16 h post-infection as described in the Materials and Methods section. Data are expressed as % of the maximum cAMP level that was measured in this experiment.

Immunoblot experiments similar to the experiments described in the previous section were also carried out infecting human epithelial HeLa cells. Since these cells are non-phagocytic, bacteria were in invasive conditions (grown overnight in LB with 0.3 M NaCl without shaking) for these experiments. The presence of SteA-3xFLAG in the pellet and the eukaryotic cytosol was analyzed 50 min and 4.5 h post-infection with anti-FLAG antibodies (Fig. 5A). Translocation to the host cytosol was observed only in the wild-type background 4.5 h post-infection. Results with anti-GroEL, indicating that this method was not sensitive enough to detect bacterial proteins 50 min post-infection, prompted us to use the CyaA system described above to study translocation into HeLa cells. *S. enterica* serovar Typhimurium wild-type strain 14028 and isogenic *prgH* mutant, and *ssaV* mutant were used to infect HeLa cells under invasive conditions and the translocation of SteA-CyaA' was monitored 15 min, 50 min, 2 h, 4 h, 8 h and 16 h post-infection. As seen in Fig. 5B, translocation from the wild-type strain was detected at the same level from 15 min to 16 h post-infection. Translocation of SteA into HeLa cells was dependent on the T3SS1, since infections with the *prgH* mutant gave low levels of cAMP. Surprisingly, the *ssaV* mutation significantly reduced translocation 15 min and 50 min post-infection, suggesting a role for T3SS2 at this short time.

### Expression of steA and the T3SS2 genes during HeLa cells infections

**Figure 6:**
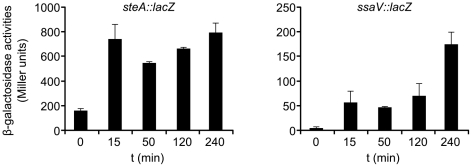
Expression of *steA* and the T3SS2 during infection of HeLa cells. HeLa cells were infected with *S. enterica* serovar Typhimurium strains carrying a *steA::lacZ* fusion (left panel) or a *ssaV::lacZ* fusion (right panel) under invasive conditions. β-galactosidase activities were measured before infection (t = 0) and 15, 50, 120, and 240 min after infection.

The results obtained in the previous section prompted us to analyze the expression of *steA* and *ssaV* (as an indicator of the synthesis of the T3SS2) in the context of the infection of HeLa cells. Derivatives of the strain 14028 of *S. enterica* serovar Typhimurium carrying *steA::lacZ* or *ssaV::lacZ* fusions, were grown under SPI-1 inducing conditions (LB 0.3 M NaCl, low aeration). Under these conditions, expression of *steA* was high although not maximal, as seen above. Under the same conditions, as expected, *ssaV* was not expressed (less than 6 Miller units). Interestingly, very quickly after infection, expression increased three-fold for *steA* and ten-fold for *ssaV* (Fig. 6), giving support to the involvement of T3SS2 in the translocation of SteA at the beginning of the infection of HeLa cells under invasive conditions.

### Identification of secretion signals in SteA

**Figure 7:**
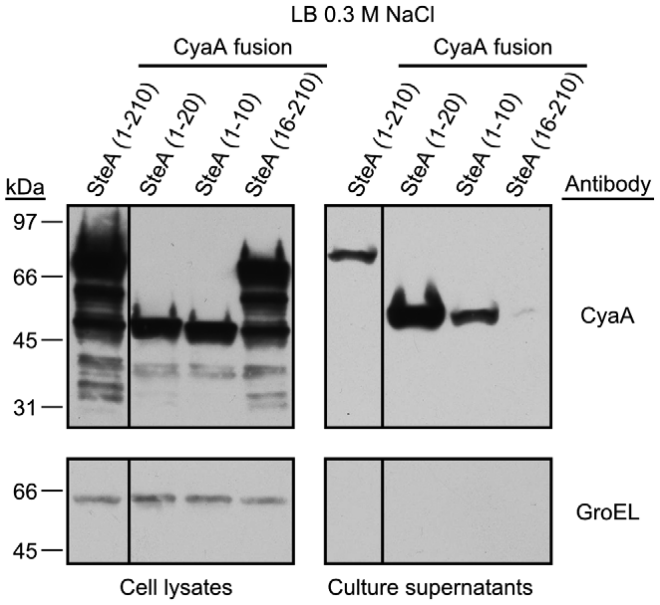
Identification of the sequences necessary for secretion of SteA. Derivatives of *S. enterica* serovar Typhimurium strain 14028 carrying plasmids expressing full-length (1–210) or different fragments of SteA in fusion with CyaA from *B. pertussis* (numbers in brackets indicate aminoacids included) were grown under SPI-1 inducing conditions. The presence of the CyaA fusions in cell lysates (from 10^8^ cells) and culture supernatants (from 2.5×10^8^ cells) was analyzed by immunoblotting with anti-CyaA antibodies as described in the Materials and Methods section (upper panels). Anti-GroEL antibodies were used as loading control and to test unspecific leakage of proteins into the supernatants (lower panels). Molecular mass markers, in KDa, are indicated on the left.

In order to investigate the signals relevant for secretion of SteA to the culture media, we generated several CyaA fusions expressed from plasmids. Different N- and C-terminal truncations were obtained and tested by immunoblotting with anti-CyaA antibodies. Fig. 7 shows the results obtained under SPI-1-inducing conditions. Fusions with the full-length protein and with fragments 1–20 and 1–10 were efficiently secreted. In contrast, the fusion with the amino acids 16–210 of SteA was not secreted. These results suggest that the signal necessary for secretion is contained in the first 10 amino acids of SteA.

### Analysis of translocation of SteA fragments into macrophages

The different CyaA fusions used in the previous section for the secretion experiments were also tested in experiments of translocation into RAW264.7 cells. The results (data not shown) were consistent with the results of the secretion experiments and suggested that the first 10 amino acids of SteA were also sufficient for translocation of this effector into the host cell.

**Figure 8:**
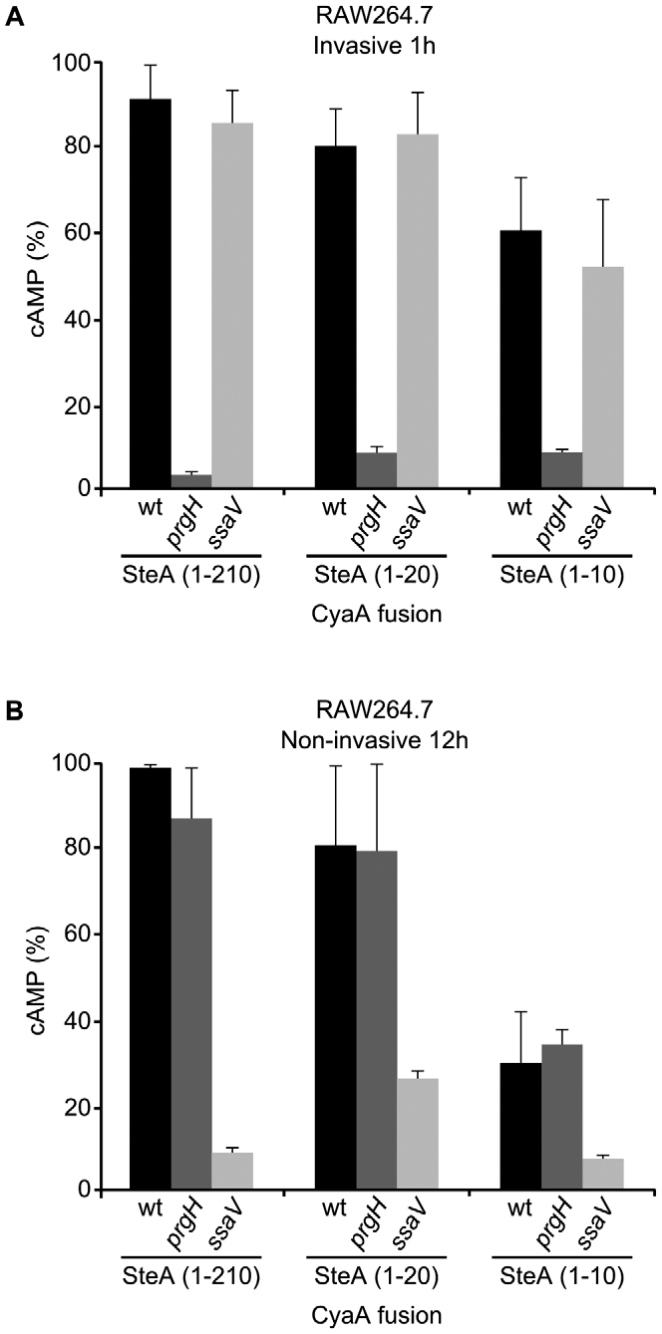
Analysis of the translocation of fragments of SteA under invasive and non-invasive conditions. RAW264.7 cells grown in 24-well plates were infected with the wild-type, *prgH*, or *ssaV* strains of *S. enterica* serovar Typhimurium expressing full-length SteA (1–210) or SteA fragments 1–10 or 1–20 in fusion with the catalytic domain of CyaA from *B. pertussis*. (A) Bacteria were grown for 16 h in LB with 0.3 M NaCl at 37°C without shaking and cAMP was measured 1 h post-infection. The results are expressed as % of the maximum level of cAMP measured in the experiment. (B) Bacteria were grown for 24 h in LB at 37°C with shaking and cAMP was measured 12 h post-infection. The results are expressed as % of the maximum level of cAMP per bacterium measured in the experiment.

*Salmonella* can enter phagocytic cells, like macrophages RAW264.7, by two different mechanisms: invasion and phagocytosis. Whereas invasion is T3SS1-dependent, phagocytosis is carried out without the participation of any T3SS. Therefore, different bacterial growth and infection conditions can be used to monitor the relevance of T3SS1 and T3SS2 in the translocation of SteA into phagocytic cells. Translocation of the CyaA fusions that were secreted to the medium (Fig. 7) and translocated into RAW264.7 (data not shown) was assessed in the mutants *prgH* and *ssaV* that are T3SS1- and T3SS2-defective strains, respectively. Translocation of the three fusions measured 1 h after infection of macrophages with invasive bacteria was T3SS1-dependent (Fig. 8A). Translocation of the same fusions measured 12 h after infection of macrophages with non-invasive bacteria was T3SS2-dependent (Fig. 8B). These results suggest that the same N-terminal motif is recognized by both *Salmonella* T3SS.

## Discussion

For an efficient secretion, the synthesis of one effector should be coordinated with the expression of its cognate T3S apparatus. Examples of this coordination are found in *Salmonella enterica* [36], [37], *Shigella flexneri* [38], [39], [40], and *Yersinia enterocolitica* [41].

This work focuses on the study of SteA, a *Salmonella* effector that was poorly characterized. It was described in 2005 as being able to translocate into macrophages J774 through T3SS1 and T3SS2 [24]. Since SteA is a *Salmonella* effector that can be secreted through two different T3SS, it was especially interesting to study expression, secretion and translocation of this effector under different conditions favoring the use of one or the other T3S apparatus.

Our results (Fig. 1 and Fig. 2) indicate that *steA* is expressed under a wide range of conditions. This is consistent with the ability of this effector to be secreted through two systems that are expressed in different conditions. However, we have identified particular factors that increase or decrease expression. A comparison of different media showed that, in vitro, optimal expression is obtained in LPM at pH 5.8, a media that imitates the conditions found in the SCV and that is also optimal for the synthesis of T3SS2. Expression of *steA*, however, was not significantly affected by changes in pH between 5.0 and 8.4 in LB with 0.3 M NaCl without aeration (Fig. 1D) or in LPM (Fig. 1E). Bacteria grown in LB media with low aeration and high osmolarity until the end of the exponential growth phase are maximally invasive due to optimal expression of T3SS1. These conditions however are not the best for expression of *steA* because, although low aeration increases expression of this gene (Fig. 1D and E), hyperosmolarity does the opposite (Fig. 1B and C). This is not just a consequence of osmotic shock since the results were obtained in the presence of proline as osmoprotector. In addition, immunoblot experiments (Fig. 2) revealed that low osmolarity also decreases the amount of SteA, in spite of the expression of the translational *steA-lacZ* fusion. This result suggests that hypoosmolarity compromises the stability of the protein SteA. The suboptimal expression of SteA in the media that provides optimal invasivity may not be totally representative of the situation during the infection, since culture media are not able to perfectly mimic the in vivo conditions. In fact, our experiments measuring the level of expression of *steA* in the course of the infection of HeLa cells suggest that this expression significantly increases upon contact of *Salmonella* with the host cell and remains elevated several hours post-infection (Fig. 6).

Another factor that decreases expression of *steA* is butyric acid (Fig. 1 and Fig. 2). This is one of the organic acids that are necessary to maintain the normal status of the intestinal epithelium [42]. In fact, treatment with butyric acid decreases invasion and colonization of cecal epithelial cells [43], [44]. In a transcriptomic analysis 19 genes were found to be down-regulated with butyrate more than two-fold in *S.enterica* serovar Typhimurium and *S. enterica* serovar Enteritidis, 17 of these genes localized to SPI-1 and one of the other two genes was *sopE2*, a gene encoding a T3SS1 effector located outside the island [45]. In addition, the level of SipA, another effector encoded outside SPI-1, is also reduced after exposition to butyrate [46]. Therefore, in regard to butyrate, *steA* behaves like many T3SS1 related genes.

We used culture media that are optimal for expression of either T3SS1 (LB 0.3 M NaCl) or T3SS2 (LPM pH 5.8) to characterize the secretion of SteA through these systems (Fig. 3). Efficient secretion was detected in both media and the secretion was dependent on the expected T3SS. It has been suggested that the complex SsaM-SpiC-SsaL regulates secretion through T3SS2 so that translocon proteins are secreted first and, once the translocon pores has been assembled and the pH of the cytosol is detected, the secretion of effectors begins [32]. As a consequence the effector SseJ is not secreted to the culture media unless the experiment is carried out in an *ssaL* background or the pH is shifted to 7.2 at the end of the experiment. This effect has also been observed for other effectors [25]. Our results, however, show that SteA can be efficiently secreted in LPM at pH 5.8 in a wild-type background, although a small increase in secretion can be observed in the *ssaL* mutant.

T3SS1 is expressed at the beginning of the infection and is important for invasion. T3SS2 is expressed inside the *Salmonella* containing vacuole and is necessary for create and maintain an intracellular niche for survival and proliferation of the bacteria. However, as mentioned in the Introduction, some overlap exists between both systems. Our experiments studying the kinetics of translocation of SteA into eukaryotic host cells provide two additional examples of this overlapping. First, non-invasive *Salmonella* translocated SteA into RAW264.7 cells mainly through T3SS2 and this is clearly seen 12 h post-infection when the *ssaV* mutation completely abolishes translocation (Fig. 4A). However, although translocation 6 h post-infection is mainly T3SS2-dependent, some translocation is observed in the *ssaV* mutant. That this translocation occurs through T3SS1 is confirmed because the double mutant shows no translocation (Fig. 4B). These results suggest that T3SS1 is expressed and is functional in the translocation of effectors inside macrophages for a certain period of time. The second example is observed in the experiments of translocation into HeLa cells using invasive bacteria. To interpret these experiments it should be taken into account that these cells are non-phagocytic. Therefore, entry of *Salmonella* depends on bacterial factors, mainly on the invasivity provided by T3SS1. This is consistent with the results of the kinetics of translocation into HeLa cells (Fig. 5B) showing that translocation is T3SS1-dependent throughout the experiment, from 15 min to 16 h. In this context it is difficult to ascertain the role of T3SS2, if any, in the translocation of effectors. Translocation for 4 h, 8 h and 16 h is measured for bacteria inside de host cells, since treatment with the antibiotic gentamicin is used to kill extracellular cells. Therefore, the T3SS1-deficient mutant *prgH*, that cannot enter the host cell, would not show translocation even if the secretion occurred through a different system. For shorter times, up to 2 h, extracellular bacteria are present in the protocol we used (see Materials and Methods), and we can detect translocation from outside the host cell through the cytoplasmic membrane. Interestingly, we observed a preponderant role for T3SS2 in the translocation of SteA into HeLa cells 15 min after the infection that decreases 50 min post-infection and is no longer detected 2 h post-infection (Fig. 5B). This result was unexpected for two reasons:

- The bacteria were grown under invasive conditions that prevent expression of T3SS2 genes. This repression was confirmed using an *ssa V::lacZ* fusion (Fig. 6, right panel, time 0). However, as seen in Fig. 6, the expression of this fusion increased very quickly during infection of Hela cells, which is consistent with the translocation of SteA through this system at the beginning of the infection. It has been previously shown that SPI-1 and SPI-2 genes are expressed simultaneously inside HeLa cells [27]. It should be noted, however, that the shorter time after infection analyzed in that report was 2 h. Here, we show that *ssaV* is expressed as early as 15 min after infection of HeLa cells. This result increases a growing body of evidence showing expression of SPI-2 immediately after the entry of the bacteria into the small intestine and indicating that T3SS2 contributes to intestinal colonization [47], [48], [49], [50]. A very recent report suggests that expression of SPI-2 before invasion of host cells reflects transcriptional priming that is needed later for intracellular survival [51]. Our results suggest that T3SS2 is not only expressed but also functional in the secretion of at least the effector SteA at that time.
- Even if 15 min post-infection SteA can be translocated through T3SS2, it is expected to be translocated also through T3SS1. The kinetics of SteA translocation in the *ssaV* single mutant suggested an irrelevant or minor contribution of T3SS1 to translocation of SteA 15 min post-infection, although translocation through T3SS1 increased with the time (Fig. 5B, compare 15 min, 50 min, and 2 h). A feature of T3SS is their ability to engage substrates in a certain order [52], [53], [54], [55], [56], [57], [58], [59]. A sorting platform has been recently described for T3SS1 that ensures secretion of the translocases before the effectors [60]. This hierarchy of secretion could also extend to the establishment of an order of secretion between effector proteins based on differential affinities of the different effector-chaperone complexes. In this context, our results suggest that SteA is secreted through T3SS1 at a relatively late stage in the secretion process. Another factor that can contribute to a low level of secretion of SteA through T3SS1 at the beginning of the infection is the fact that this protein, unlike other T3SS1 effectors, is also a substrate of T3SS2.

In should be noted, however, that in the conditions discussed above the detection of SteA in the host cytosol was also dependent on T3SS1. This result could be a consequence of the role of this system not only in translocation of proteins but also in host cell binding [56], [61].

Due to low sequence similarity and lack of common features among different T3S signal sequences, attempts to use computational methods to identify these signals have only achieved limited accuracy [62], [63], [64], [65], [66]. As a result, experimental approaches based on the generation of fusions with truncated forms of effector proteins are necessary. Previous studies with other effectors have shown that some kind of secretion signal exists at the N-terminus [67], [68], [69], [70], [71]. In addition, for T3SS1 effectors like InvJ and SipB small sequences contained in the N-terminal 10 amino acids are enough to mediate secretion through this system [72], [73]. Our experiments with truncated forms of SteA show that the N-terminal 10 amino acids of this protein are sufficient to direct secretion into culture media (Fig. 7) and translocation into eukaryotic host cells (Fig. 8) although the N-terminal 20 amino acids are necessary for optimal secretion and translocation, in particular through the T3SS2.

Finally, the results obtained with *prgH* and *ssaV* mutants in macrophages suggest that the same N-terminal signal is recognized by T3SS1 and T3SS2 (Fig. 8). These results also support the idea of the universality of the secretion signals recognized by these systems. In fact, the ability of effectors from one species to be translocated by the T3SS of another bacterial species has been documented [74], [75], [76]. In addition, it has been shown that the N-terminal secretion sequence of the *Salmonella* T3SS1 effectors SopE and SptP can direct secretion through the related flagellar system [77]. In these cases, translocation through T3SS1 requires a chaperone-binding domain, located in the first 140 amino acids, in addition to the N-terminal secretion sequence. Although the existence of a similar chaperone mechanism for SteA cannot be ruled out, our experiments show that the N-terminal signal located in the first 10 amino acids is enough to direct specific translocation into RAW264.7 cells through T3SS1 and T3SS2. Additional experiments are needed to identify chaperones for SteA and the cognate chaperone-binding domain in the effector, if they exist.

## Materials and Methods

### Bacterial strains, bacteriophages and strain construction

*E. coli* and *S. enterica* serovar Typhimurium strains used in this study are described in Table 1. *Salmonella* strains derive from the mouse-virulent strain ATCC 14028. Transductional crosses using phage P22 HT 105/1 *int201* [78] were used for strain construction [79]. To obtain phage-free isolates, transductants were purified by streaking on green plates [80]. Phage sensitivity was tested by cross-streaking with the clear-plaque mutant P22 H5.

**Table 1:** Bacterial strains and plasmids used in this study.

Strain/Plasmid	Relevant characteristics	Source/Reference
*E. coli*		
DH5α	*supE44* Δ*lacU*169 (Ø80 *lacZ*ΔM15) *hsdR17 recA1 endA1 gyrA96 thi-1 relA1*	[87]
TP610	*F-, thi-1 thr-1 leuB6 lacY1 tonA21 supE44 hsdR hsdM recBC lop-11 lig^+^cya-610*	[88]
*S. enterica* serovar Typhimurium		
14028	Wild type	ATCC
SV5846^a^	14028 *steA*::3xFLAG, Km^r^	This study
SV6152	14028 *steA::lacZ*	This study
SV5379	14028 Δ*prgH*	Laboratory stock
SV5136	14028 *ssaV::*Cm^r^	Laboratory stock
SV5604	14028 Δ*prgH ssaV::*Cm^r^	Laboratory stock
*Plasmids*		
pKD13	*bla* FRT *aph* FRT PS1 PS4 oriR6K	[83]
pKD46	*bla* PBAD *gam bet exo* pSC101 oriTS	[83]
pCP20	*bla cat cI*857 λP_R_ *flp* pSC101 oriTS	[89]
pCE40	*aph* FRT *‘lacZ lacY^+^* t*_his_* oriR6K	[84]
pSIF003-R1	pEX-CyaA_1–412_ derivative	[81]
pIZ1673	pSIF003-R1 Δ*lacI*	This study
pIZ1886	pIZ1673-SteA	This study
pIZ1889	pIZ1673-SteA(1–20)	This study
pIZ1890	pIZ1673-SteA(1–10)	This study
pIZ1891	pIZ1673-SteA(16–210)	This study

a Derivatives of these strains were used as indicated in the text.

### Bacterial culture

The standard culture medium for *S. enterica* was Luria-Bertani (LB) broth. Solid LB contained agar 1.5% final concentration. Antibiotics were used at the following concentrations: kanamycin (Km), 50 µg ml^−1^; chloramphenicol (Cm), 20 µg ml^−1^; ampicillin (Ap), 100 µg ml^−1^. For SPI-1-inducing conditions, *Salmonella* strains were grown overnight at 37°C in LB-0.3M NaCl medium in static conditions. For SPI-2-inducing conditions, cells from cultures in LB were washed and diluted 1∶100 with minimal medium at pH 5.8 (LPM) containing 80 mM 2-(N-morpholino) ethanesulfonic acid (pH 5.8), 5 mM KCl, 7.5 mM (NH_4_)_2_SO_4_, 0.5 mM K_2_SO_4_, 0.1% casamino acids, 38 mM glycerol, 337.5 µM K_2_HPO_4_-KH_2_PO_4_ (pH 7.4) and 8 µM MgCl_2_, and incubated overnight at 37°C with shaking. For some experiments the concentration of NaCl or the pH of the medium were modified as indicated. 10 mM sodium butyrate (Sigma) was added to the medium in some experiments.

### DNA amplification with the polymerase chain reaction

Amplification reactions were carried out in a Perkin Elmer Gene-Amp PCR System 2400 (Perkin Elmer Cetus). The final volume of reactions was 100 µl, and the final concentration of MgCl_2_ was 1.5 mM. Reagents were used at the following concentrations: dNTPs, 300 µM; primers, 0.3 µM; and Taq polymerase (KAPA HiFi DNA Polymerase, Kapa Biosystems), 1 unit per reaction. The thermal program included the following steps: (i) initial denaturation, 5 min at 95°C; (ii) 25 cycles of denaturation (98°C, 20 s), annealing (57°C, 15 s), and extension (72°C, 30 s); and (iii) final incubation at 72°C for 5 min, to complete extension. Primers are listed in Table 2. PCR constructs were sequenced with an automated DNA sequencer (Stab Vida, Oeiras, Portugal) to confirm that the sequence was correct (no new sequencing data was generated in this work).

**Table 2:** Oligonucleotides used in this study.

Oligonucleotide/use	Sequence 5′-3′ (restriction sites are underlined)
*Insertion in steA*	
steAP1	AACGCTTTTTAATAATTGTCCAAATAGTTATGGTAGCGAGGTGTAGGCTGGAGCTGCTTC
steAP4	TACTATAGATGTCGAGCTTTCTGATGGTCGGATGTTAACAATTCCGGGGATCCGTCGACC
*Epitope tagging of SteA*	
steAP1flag	CGACATAAAAGCTCGCTACCATAACTATTTGGACAATTATGACTACAAAGACCATGACGG
steAP2flag	AGTCTGATTTCTAACAAAACTGGCTAAACATAAACGCTTTCATATGAATATCCTCCTTAG
*Construction of pIZ1886*	
steAbampsif5′	GAATGGATCCAGGAGGTAGGATATGCCATATACATCAGTTTC
steAbampsif3′	CAAGGGATCCAATAATTGTCCAAATAGTTATG
*Construction of pIZ1889*	
steAbampsif5′	as above
steA20bampsif3′	CAAGGGATCCAATGAGGTAGCTTATTTCCTG
*Construction of pIZ1890*	
steAbampsif5′	as above
steA10bampsif3′	CAAGGGATCCAGGCATAGGTAGAAACTGATG
*Construction of pIZ1891*	
steA16bampsif5′	GAATGGATCCAGGAGGTCGACTATGAATAAGCTACCTCATGTTG
steAbampsif3′	as above
*Construction of pIZ1892*	
steA31bampsif5′	GAATGGATCCAGGAGGTCGACTATGTCAACAAAAATCATGAAAG
steAbampsif3′	as above

### Plasmids

Plasmids used in this study are listed in Table 1. Plasmids expressing CyaA' fusions were derivatives of pIZ1673, a modification of pSIF003-R1 [81] (a gift from I. Rosenshine, the Hebrew University of Jerusalem) that was constructed by deletion of the *lacI* gene using enzymes *Hpa*I and *Sph*I. Therefore, expression of CyaA fusions from derivatives of pIZ1673 is no longer dependent on IPTG. To construct these plasmids, DNA from strain 14028 was used as a template for PCR amplification with the primers listed in Table 2. The amplified fragments were digested with *Bam*HI and ligated with *Bam*HI-digested and dephosphorylated pIZ1673. To detect successful constructs (plasmid with insert in the correct orientation), the ligation mixture was transformed into *E. coli* TP610 and transformants were selected in MacConkey agar supplemented with Ap and with 1% maltose. TP610 is a *cya* mutant strain [82] that can be complemented with the basal adenylate cyclase activity that possesses CyaA from *B. pertussis* in the absence of calmodulin. Since *cyaA* in pIZ1673 does not harbor a start codon for translation, transformants with empty plasmids or with inserts in the wrong orientation give white colonies in MacConkey-maltose, whereas plasmids with inserts in the correct orientation give red colonies in the same medium.

### Mammalian cell culture

HeLa cells (human epithelial; ECAC no. 93021013) and RAW264.7 cells (murine macrophages; ECACC no. 91062702) were cultured in DMEM supplemented with 10% foetal calf serum. 2 mM L-glutamine, 100 U ml^−1^ penicillin, and 100 µg ml^−1^ streptomycin were included in the culture media. All cells were maintained in a 5% CO_2_ humidified atmosphere at 37°C.

### Construction of a chromosomal lacZ fusion

Disruption and replacement of *steA* with a Km resistance gene were performed as described previously [83]. Briefly, the Km resistance gene from plasmid pKD13 was PCR amplified with primers steAP1 and steAP4. The sequences of the primers used are shown in Table 2. The PCR product was used to transform the wild-type strain carrying the Red recombinase expression plasmid pKD46. The antibiotic resistance cassette introduced by the gene-targeting procedure was eliminated by recombination using the FLP helper plasmid pCP20 [83]. The FRT site generated by excision of the antibiotic resistance cassette was used to integrate plasmid pCE40 to generate a translational *lac* fusion [84].

### Chromosomal gene epitope tagging

Addition of a DNA fragment encoding the 3xFLAG epitope tag at the 3′ end of *steA* was carried out as described [85] using primers SteA-P1Flag and SteA-P2Flag.

### Western blotting and antibodies

*Salmonella* strains were grown under different conditions. Usually, cultures in LB medium were diluted and grown in different media. The bacteria were then pelleted by centrifugation and resuspended in sodium dodecyl sulfate-polyacrylamide gel electrophoresis (SDS-PAGE) sample buffer. Proteins from the same numbers of bacteria were separated by gradient SDS-PAGE (Mini-PROTEAN TGX precast gels, 4–15%) and electroforetically transferred to nitrocellulose filters for Western blot analysis using anti-Flag M2 monoclonal antibodies (1∶5000; Sigma), anti-GroEL polyclonal antibodies (1∶20000; Sigma) or anti-CyaA (3D1) monoclonal antibodies (1∶1000; Santa Cruz Biotechnology). Goat anti-mouse HRP-conjugated antibodies (1∶5000; BioRad) and goat anti-rabbit HRP-conjugated antibodies (1∶10000; GE Healthcare) were used as secondary antibodies.

### Protein secretion analysis

*Salmonella* strain SV5846 (14028 expressing SteA-3xFLAG) and *prgH* or *ssaV* derivatives were grown in the different media described above. For some experiments, strain 14028 carrying plasmids pIZ1886, pIZ1889, pIZ1890, or pIZ1891, expressing different fragments of SteA in fusion with CyaA from *B. pertussis*, were grown under SPI-1-inducing conditions (LB 0.3 M NaCl, overnight, without shaking). Whole cells and culture supernatants were separated by centrifugation at 13000 g for 10 min. The supernatants were filtered (0.45 µm pore-size) and incubated 30 min on ice with 0.02% deoxycholate. Proteins were then precipitated by adding trichloroacetic acid at a final concentration of 10% v/v, followed by incubation at −20°C for 5 min, on ice for 15 min and centrifugation (13000 g, 4°C, 15 min). The supernatant was discarded and the pellet was incubated with 300 µl of cold acetone, 15 min on ice. After a new centrifugation step, the pellet and the whole cells were processed for electrophoresis and Western blot.

### Bacterial infection of cultured cells

HeLa or RAW 264.7 cells were plated in 24-well plates at 1.5×10^5^ cells per well and incubated 24 h at 37°C with 5% CO_2_. For the infection of HeLa cells under SPI-1 (invasive) conditions, bacteria grown overnight in LB-0.3M NaCl in a tightly closed tube without shaking were added at a multiplicity of infection (MOI) of 150. The same conditions were used for infections of macrophages RAW264.7 under invasive conditions except that the MOI was 50. For infections of RAW264.7 under non-invasive conditions, bacteria were grown in LB for 24 h at 37°C with shaking and were added at a MOI of 250. Bacteria were centrifuged onto the cell monolayer at 200 g for 5 min and then incubated at 37°C with 5% CO_2_ for 15 min to 2 h. For longer infections, the cell culture was washed twice with phosphate-buffered saline (PBS) 1 h post-infection, overlaid with DMEM containing 100 mg ml^−1^ gentamicin, and incubated for another hour. The culture was then washed twice with PBS, DMEM with gentamicin 16 mg ml^−1^ was added, and the culture was incubated for another 2 to 14 h.

### β-Galactosidase assays

Levels of β-galactosidase activity were assayed for stationary phase cultures in LB medium or in minimal medium as described [86], using the CHCl_3_/SDS permeabilization procedure. Enzymatic assays were also carried out during infection of HeLa cells. The cell culture, infected as described above, was washed twice with PBS, lysed with 1% Triton X-100 in 100 µl of PBS to release intracellular bacteria. 100 µl of PBS were added and the whole lysate was used for the β-galactosidase assay. The number of bacteria in the assay was determined by plating serial dilutions from a duplicate experiment and enumerating the colonies on the following day. In order to calculate conventional Miller units, the number of colony forming units was converted into the corresponding value of OD600. This correspondence was obtained by calculating OD600 and number of colony forming units in the bacterial culture used in the infection. The β-galactosidase activity of this culture was also measured and used as time t = 0 in the experiment.

### Protein translocation assays

Following the infections described above, the translocation of SteA fusions into the eukaryotic cells was monitored either by immunoblot or by measuring the levels of cAMP. To study translocation of 3xFLAG fusions, infected mammalian cells were lysed with 0.1% Triton X-100. Cell lysates were centrifuged at 13000 g for 20 min at 4°C to separate the soluble fraction, consisting of the host cell cytosol and translocated bacterial proteins, from the insoluble fraction, containing the internalized bacteria. The soluble fraction was filtered through a 0.22 µm pore-size filter and subjected to TCA precipitation as described above. Samples from both fractions were subjected to SDS-PAGE and immunoblot using anti-FLAG antibodies. To study translocation of CyaA fusions, the infected cells were lysed and the level of cAMP in the lysates were determined using a colorimetric direct cAMP enzyme immunoassay kit (Arbor Assays) according to the manufacturer's instructions. For experiments in RAW 264.7 macrophages infected for 6 h or 12 h, the levels of cAMP were normalized to the number of intracellular bacteria. Data are represented as % of the maximum level of cAMP that was measured in the experiment.
